# Prediction of binding property of RNA-binding proteins using multi-sized filters and multi-modal deep convolutional neural network

**DOI:** 10.1371/journal.pone.0216257

**Published:** 2019-04-26

**Authors:** Taesu Chung, Dongsup Kim

**Affiliations:** Department of Bio and Brain Engineering, Korea Advanced Institute of Science and Technology, Daejeon, Republic of Korea; Newcastle University, UNITED KINGDOM

## Abstract

RNA-binding proteins (RBPs) are important in gene expression regulations by post-transcriptional control of RNAs and immune system development and its function. Due to the help of sequencing technology, numerous RNA sequences are newly discovered without knowing their binding partner RBPs. Therefore, demands for accurate prediction method for RBP binding sites are increasing. There are many attempts for RBP binding site predictions using various machine-learning techniques combined with various RNA features. In this work, we present a new deep convolution neural network model trained on CLIP-seq datasets using multi-sized filters and multi-modal features to predict the binding property of RBPs. With this model, we integrated sequence and structure information to extract sequence motifs, structure motifs, and combined motifs at the same time. The RBP binding site prediction on RBP-24 dataset was compared with two multi-modal methods, GraphProt and Deepnet-rbp, using area under curve (AUC) of receiver-operating characteristics (ROC). Our method (average AUC = 0.920) outperformed 20 RBPs with GraphProt (average AUC = 0.888) and 15 RBP with Deepnet-rbp (average AUC = 0.902). The improvement was achieved by using multi-sized convolution filters, where average relative error reduction was 17%. By introducing new RNA structure representation, structure probability matrix, average relative error was reduced by 3% when compared to one-hot encoded secondary structure representation. Interestingly, structure probability matrix was more effective on ALKBH5, where relative error reduction was 30%. We developed new sequence motif enrichment method, which we stated as response enrichment method. We successfully enriched sequence motif for 12 RBPs, which had high resemblance with other literature evidences, RBPgroup and CISBP-RNA. Finally by analyzing these results altogether, we found intricate interplay between sequence motif and structure motif, which agreed with other researches.

## Introduction

RNA-binding proteins (RBPs) are important in many biological activities by post-transcriptional control of RNAs. The roles of RBPs are diverse; mRNA expression controlling (translation initiation, elongation, 5`-capping, methylation, and polyadenylation), protein translation controlling (transcription, splicing, and decaying), mRNA exportation from various compartments inside cell and intracellular localization of RNA, gene silencing by forming RNA-induced silencing complex (RISC), and ribosome assembly [1]. RBPs even participate in immune systems by binding to specific sequence or structural motif of RNA. For example, RBPs maintain poised cytokine mRNAs for rapid translation in response to T cell receptor (TCR) signaling in memory T cells [2]. Various researches on RNA-protein interaction reported its growing complexity. A recent study on ribonucleoprotein (RNP) complexes revealed the existence of complex protein-RNA interaction which does not require canonical RNA binding domains (RBDs) such as the RNA recognition motif (RRM), hnRNP K homology domain (KH), or DEAD box helicase domain [3].

As the number of protein-binding RNAs are rapidly increasing with the help of new high throughput experimental methods such as ultraviolet-mediated cross-linking of RNA to protein *in vivo* coupled with quantitative mass spectroscopy [4–6], need for accurate computational methods which can predict RBPs with complex binding modes is also increasing. Numerous methods have been developed using combination of various information with different machine learning techniques to predict RBP binding property. GraphProt used sequence information with hypergraph representation of secondary structure information combined with support vector machine (SVM), to predict the binding sites in 24 CLIP-seq dataset [7]. DeepBind only used sequence information combined with a deep learning technique based on convolution neural network (CNN) to predict the binding sites of DNA binding proteins (DBP) and RBPs [8]. Deepnet-rbp was the first method to utilize tertiary structure information of RNA combined with sequence and structure information using flexible deep learning framework of deep belief network to predict RBP binding sites [9].

There are various issues to consider when designing prediction model. To train a prediction model, deep learning-based computational models use predefined hyper-parameters determined by numerous optimization attempts. In particular, convolution filter size used in DeepBind [8] for DBP and RBP binding prediction was fixed to 16, and the word size was fixed to 6-letters length in Deepnet-rbp [9]. However, CLIP-seq dataset curated by GraphProt contains RBP binding sites of various size ranging from 25~75 base pairs, suggesting that the protein binding sites on RNAs can differ even in a single CLIP-seq dataset. Therefore, by limiting hyper-parameters for one dataset might affect prediction capability on the other datasets. In addition, even though RNA secondary structure can have multiple forms [10], but most of the methods do not consider using multiple secondary information on a single position nor the positional information of nucleic acids forming pairwise interaction. Finally, although RBP-binding RNAs can have complex combination of sequence and structure motifs, previous researches utilized sequence and structure information separately and neglected a presence of combined motifs [7,9]. RBPs can simply bind to a specific sequence motif or a structure motif such as well-defined stem structure or hairpin loop structure [11], but they can also have complex binding modes such as RNA binding to disordered protein region by co-folding [12], interaction mediated by complex shape complementarity [13], or binding to multiple proteins with multiple RBP binding sites [3,14]. To fulfill these criteria, we decided to build a deep learning framework, which can process various sizes of RBP binding sites, use of multiple secondary structure information, and detect complex RBP binding motif.

Recently, machine-learning algorithms based on deep learning technique have demonstrated significant prediction power improvement in many different areas. For example, multiple layers of deep convolution neural network (CNN) combined with residual network showed extraordinary performance when dealing with hierarchical structural information such as image recognition [15]. Multi-modal learning by combinations of different data types using deep learning framework improved performance in the field of audio and video signal reconstructions [16]. Also, multi-modal framework was applied in various RBP binding site prediction methods. Cross-domain features and sequence information were integrated using DBN and CNN in iDeep [17]. DLPRB used CNN to transform sequence and structure information into shared representation and used recurrent neural network (RNN) to combined these features [18]. In addition, deep CNN showed good performance in many bioinformatics areas. For example, by using multiple filters combined with bidirectional long short term memory (BLSTM) layer, improvements was observed when predicting subcellular location of proteins only using sequence information [19] and even in RBP binding site prediction using sequence and structure information, iDeepS [20].

In this article, we designed a complex deep CNN, which can process two different types of data, sequence and structure information, to extract both simple and complex motifs of RBP binding sites. For deep learning framework, we implemented multiple convolution layers since higher-level RNA structures such as secondary and tertiary structures are the result of combination of lower-level structure and sequence information. For example, a hairpin loop is constructed by combination of stem and loop structure. In addition, multi-sized filters were used to extract various sized low-level motifs and applied extra convolution layers on combined motif representation for complex motif extraction. The novelties of our work are: (1) designing a deep CNN architecture by integrating sequence and structure information to achieve the state of the art prediction accuracy (2) detection of short, medium, and long motifs using multi-sized filter (3) using combined representation of sequence and structure information to extract higher-level motifs (4) introducing of filter response enrichment analysis to extract sequence, structure, and combined motifs.

## Results

**Fig 1:**
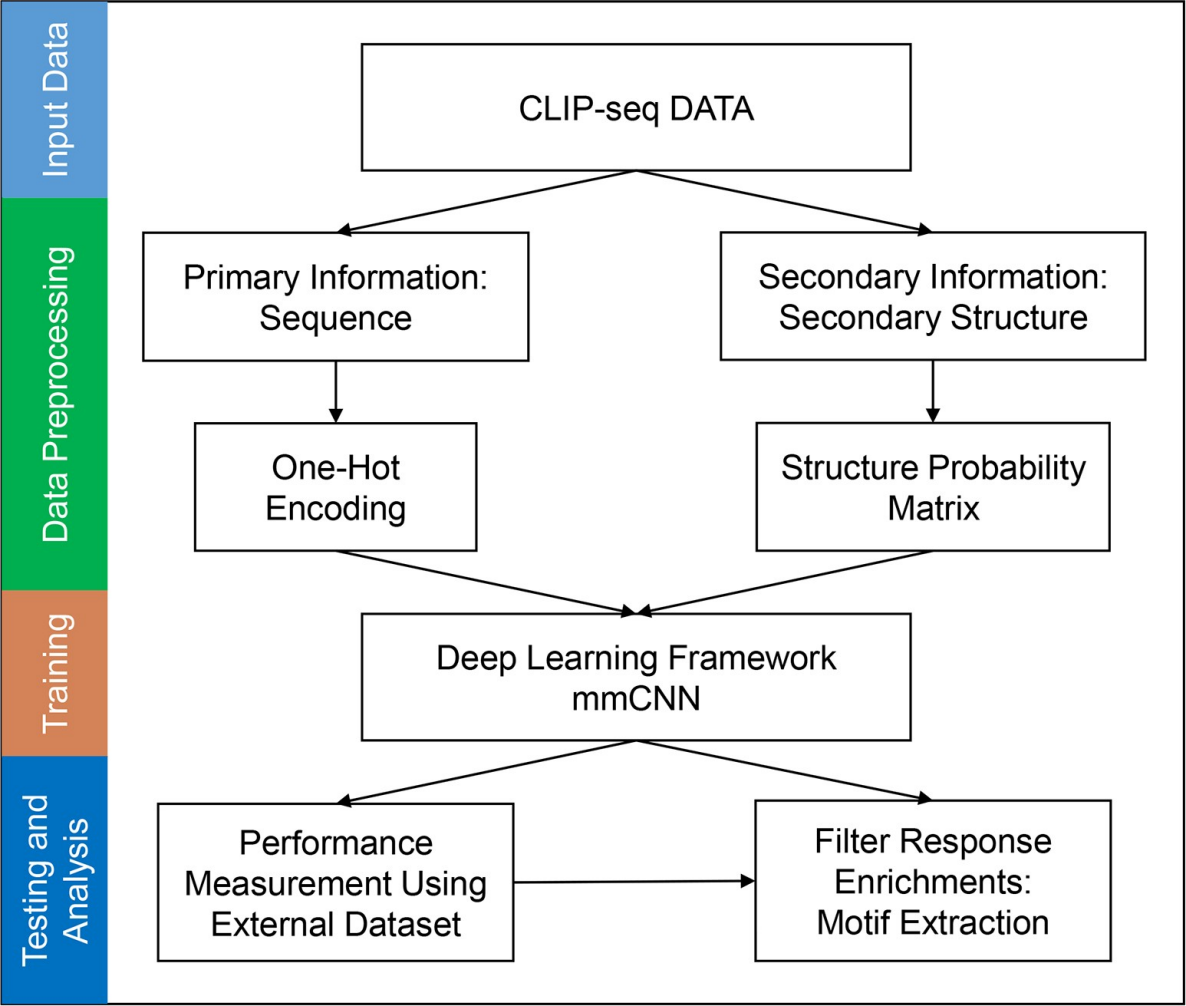
Overall summary of training procedure and performance measurement of mmCNN. Sequence data was transformed into one-hot encoded information and structure probability matrix using RNAshapes program. Bimodal information was integrated into combined representation and trained by mmCNN framework. During performance measurement, prediction of RBP binding site and its motif was predicted using external test dataset.

**Fig 2:**
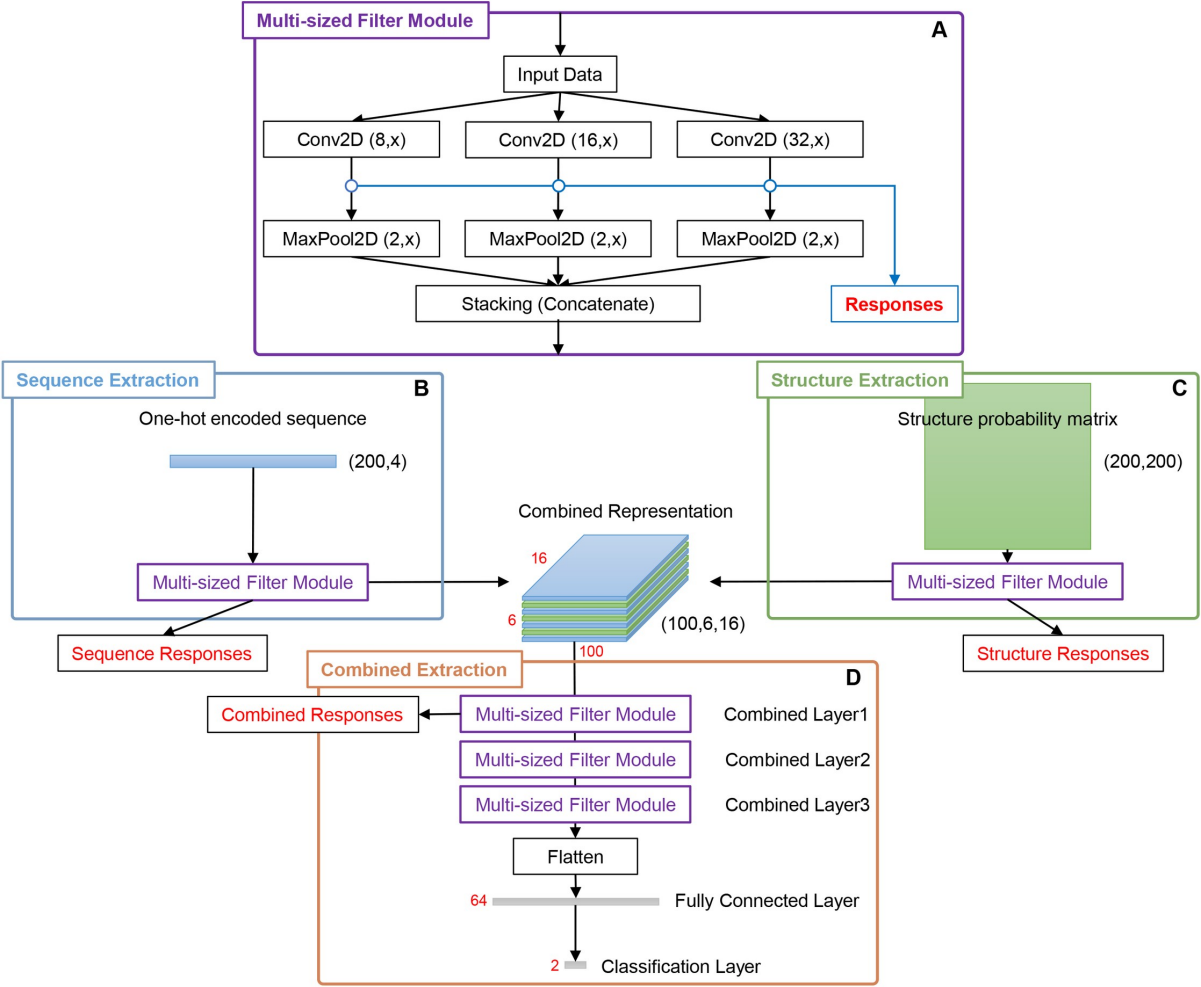
Full schematics of mmCNN architecture. (A) Multi-sized convolution module (MCM) was constructed using three different filter sizes, which were 8, 16, and 32. For convolution and max pooling operation height ***x*** and max pooling dimension *y* were used to indicate variations of values in different stages of the network. For sequence convolution ***x*** = 4 was used since sequence information was expressed in (200, 4) one-hot encoded representation. For structure convolution ***x*** = {8,16,32} were used, to capture 2-dimensional information of structure probability matrix with dimension of (200, 200). For combined convolution ***x*** is the size of number of stacked data. For max pooling operation, *y* was either 1 or 2. For max pooling in sequence and combined path *y* = 1, since convolution padding used in these paths was “valid” which shrinks height dimension into 1. Finally for max pooling in structure path, *y* = 2. (B, C) One-hot encoded sequence information and structure probability matrix passed through 1 layer of MCM. (D) Since three different sized filters were used as in (A) three stacked convolution outputs were generated for each sequence and structure input. These outputs were stacked into combined representation and went through three additional MCM layers, which were followed by max pooling, flatten and classification layer.

To study the complex motif formation by sequence and structure motifs, we developed a new multi-modal, multi-filter deep convolution neural network (mmCNN) model. The overall procedure of our research is summarized in (Fig 1). In order to train mmCNN, we first prepared a dataset for binary classification problem using CLIP-seq dataset from GraphProt [7], RBP-24 dataset. This dataset contains 24 RBPs each of which contains 1,200 ~ 125,000 sequences of positive and negative data. Preprocessed CLIP-seq data were converted into appropriate representations. RNA sequence information was transformed into one-hot encoded representation. Structure probability matrices were calculated using multiple secondary structures calculated by using RNAshapes [21]. Then, this information was used to train and optimize mmCNN using the ten-fold cross validation procedure. Performance of our models was tested on external test sets. Finally, RBP binding motifs were extracted by our newly developed motif searching method (“Methods” section). Briefly, architecture of mmCNN consists of following layers. We used two separate convolution layers to consider bi-modality of sequence and structure information (Fig 2B and 2C). Outputs of these convolution layers were stacked into a single output, and fed into three convolution layers for combined feature extraction (Fig 2D). Each convolution layer contains three filters of different size for multi-length sequence feature detection followed by rectifier linear unit (ReLU), max pooling, and dropout layers (Fig 2A). Fully-connected network with 64-nodes were used and binary cross entropy layer was added for classification. Whole network is constructed using Keras library

### General performance of mmCNN

General performance of our deep learning architecture was tested on RBP-24, which contains ICLIP, PARCLIP, and CLIP-SEQ data. For benchmarking, we compared our method with three other methods, GraphProt, Deepnet-rbp, and iDeepE. Note that GraphProt and Deepnet-rbp used both sequence and structure information, while iDeepE only used sequence information. In this article, Deepnet-rbp model using tertiary structure is denoted as DBN+, while the model not using tertiary structure as DBN-. Ten-fold cross validation procedures were performed on RBP-24, and area under curve (AUC) of receiver-operating characteristics (ROC) was used to compare overall predication power of our method with those of other methods.

Overall performance is summarized in (Table 1). When mmCNN was compared with multi-modal method only, our method was better in 20 RBPs with GraphProt and 15 RBPs with DBN+, even though we have not used tertiary structure of RNA. Notable performance improvement, greater than 10% increase when using the *relative error reduction (c’–c)/(1 –c)* where *c’* is the accuracy of new method and *c* is that of the other method [7], observed in ALKBH5, C22ORF28, AGO2, ELAVL1 (CLIPSEQ-ELAVL1), SFRS1, HNRNPC, TDP43, TIA1, TIAL1, ELAVL1(A), and IGF2BP123, which is indicated with bold case in (Table 1). Especially, prediction power of our method was notable in MOV10 and PTB, which were RBPs reported to have tertiary structure motif. Our method outperformed MOV10 with DBN+, but was less accurate when predicting PTB. RBPs with relatively small datasets such as ALKBH5, C17ORF85, and CAPRIN1 showed poor prediction results, but improvements were observed when initial weights of convolution layers were set to uniform random ranging from (0.0001, 0.01).

**Table 1:** AUROC comparison with other methods.

	GraphProt	DBN-	DBN+	mmCNN
ALKBH5	0.686	0.686	0.714	**0.766**
C17ORF85	0.817	0.817	0.820	0.829
C22ORF28	0.751	0.783	0.792	**0.865**
CAPRIN1	0.855	0.825	0.834	0.808
AGO2	0.765	0.805	0.809	**0.895**
ELAVL1	0.955	0.946	0.966	**0.980**
SFRS1	0.898	0.927	0.931	**0.942**
HNRNPC	0.952	0.961	0.962	**0.979**
TDP43	0.874	0.874	0.876	**0.927**
TIA1	0.861	0.888	0.891	**0.934**
TIAL1	0.833	0.867	0.870	**0.941**
AGO1234	0.895	0.872	0.881	0.892
ELAVL1(B)	0.935	0.956	0.961	0.960
ELAVL1(A)	0.959	0.965	0.966	**0.982**
EWSR1	0.935	0.964	0.966	0.953
FUS	0.968	0.979	0.980	0.977
ELAVL1(C)	0.991	0.994	0.994	0.991
IGF2BP123	0.889	0.872	0.879	**0.924**
MOV10	0.863	0.831	0.854	0.867
PUM2	0.954	0.965	0.971	0.967
QKI	0.957	0.981	0.983	0.974
TAF15	0.970	0.980	0.983	0.984
PTB	0.937	0.879	0.983	0.955
ZC3H7B	0.820	0.786	0.796	0.792
average	0.888	0.892	0.903	0.920

DBN-: Deepnet-rbp without tertiary structure, DBN+: Deepnet-rbp with tertiary structure, mmCNN: multi-sized filter multi-modal deep CNN. Averaged AUC of models from 10-fold cross validation was used. Performances with AUC improvement greater than 2% comparing to DBN+ were indicated in bold.

### Performance increase using multi-sized filter and structure probability matrix

**Fig 3:**
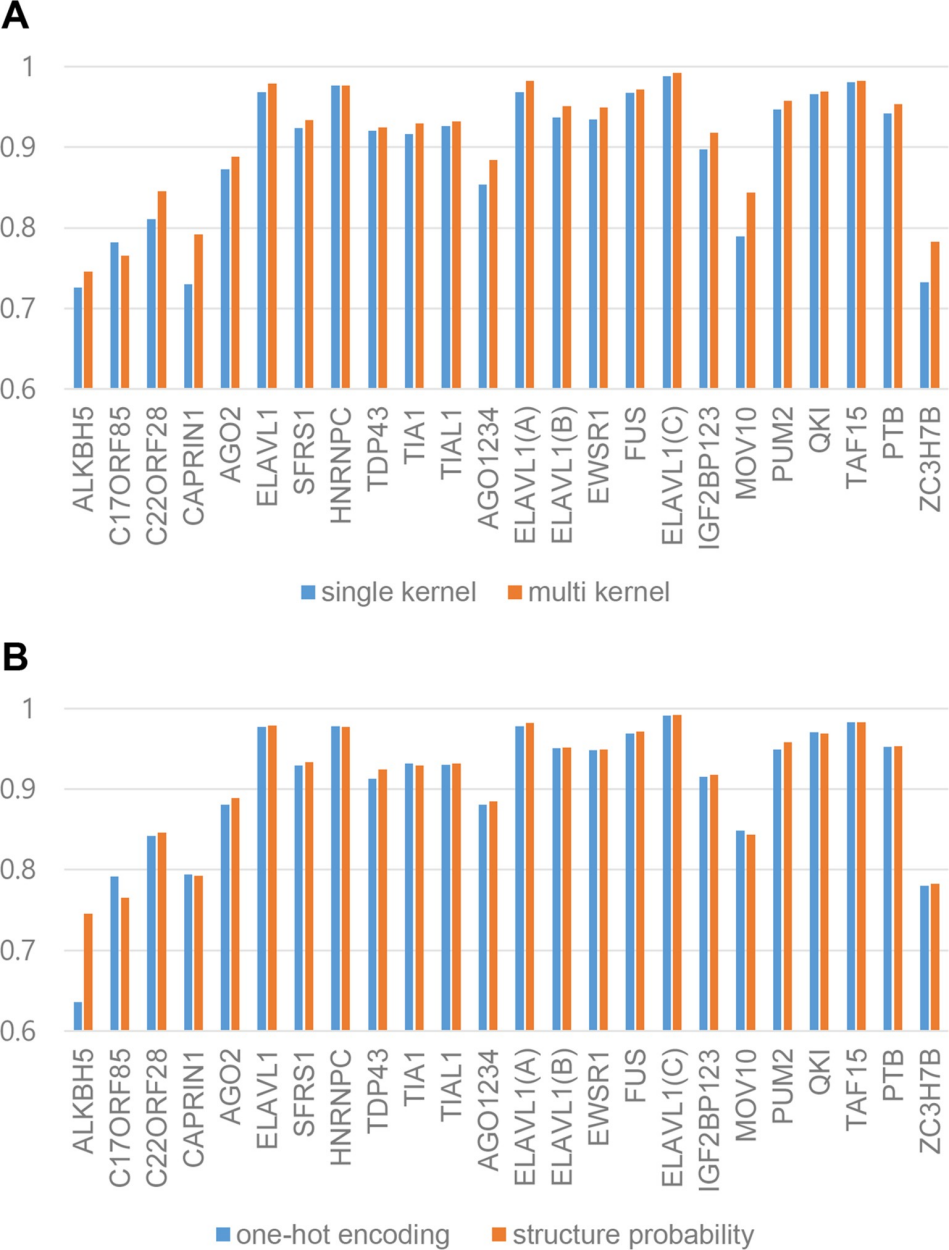
Contributions of various information in mmCNN by comparing AUC of RBP-24. (A) Comparison between single filter mmCNN and multi-sized filter mmCNN using RBP-24 dataset. (B) Better structure representation selection by comparison between one-hot encoded RNA and structure probability matrix.

The prediction performance improvement was subject to using multi-sized filters and/or structural probability matrix. In order to test these, we designed two separate experiments using several variations of the original mmCNN architecture. To test the effect of multi-sized filters, we compared performance between two models, one with the same architecture as mmCNN and the other with only using 48 filters with the size of 16 instead of 16 filters with three different sizes. By measuring relative error reduction, accuracy of multi-sized filter method was improved by 17% compared to single filter used method, (Fig 3A). To prove the advantage of using structure probability, two separate structure representations were created, structure probability matrix and one-hot encoded secondary structure which was expressed with 6-letter codes S, M, H, I, B, and E (which stands for stem, multi loop, hairpin loop, internal loop, bulge and external region) [9]. The compared results can be seen in (Fig 3B), the overall relative error reduction was improved by 4% when structure probability matrices were used. Interestingly, 30% improvement was observed in ALKBH5 whether other RBPs showed slight improvements. Additionally, we found that structure information used in mmCNN diminishes as the more convolution layers were used. This phenomenon might be natural behavior, since multiple convolution layers can capture hierarchical structures of various data. For detailed contributions of structure information, see (S1–S3 Figs).

### Sequence motif found by mmCNN

**Fig 4:**
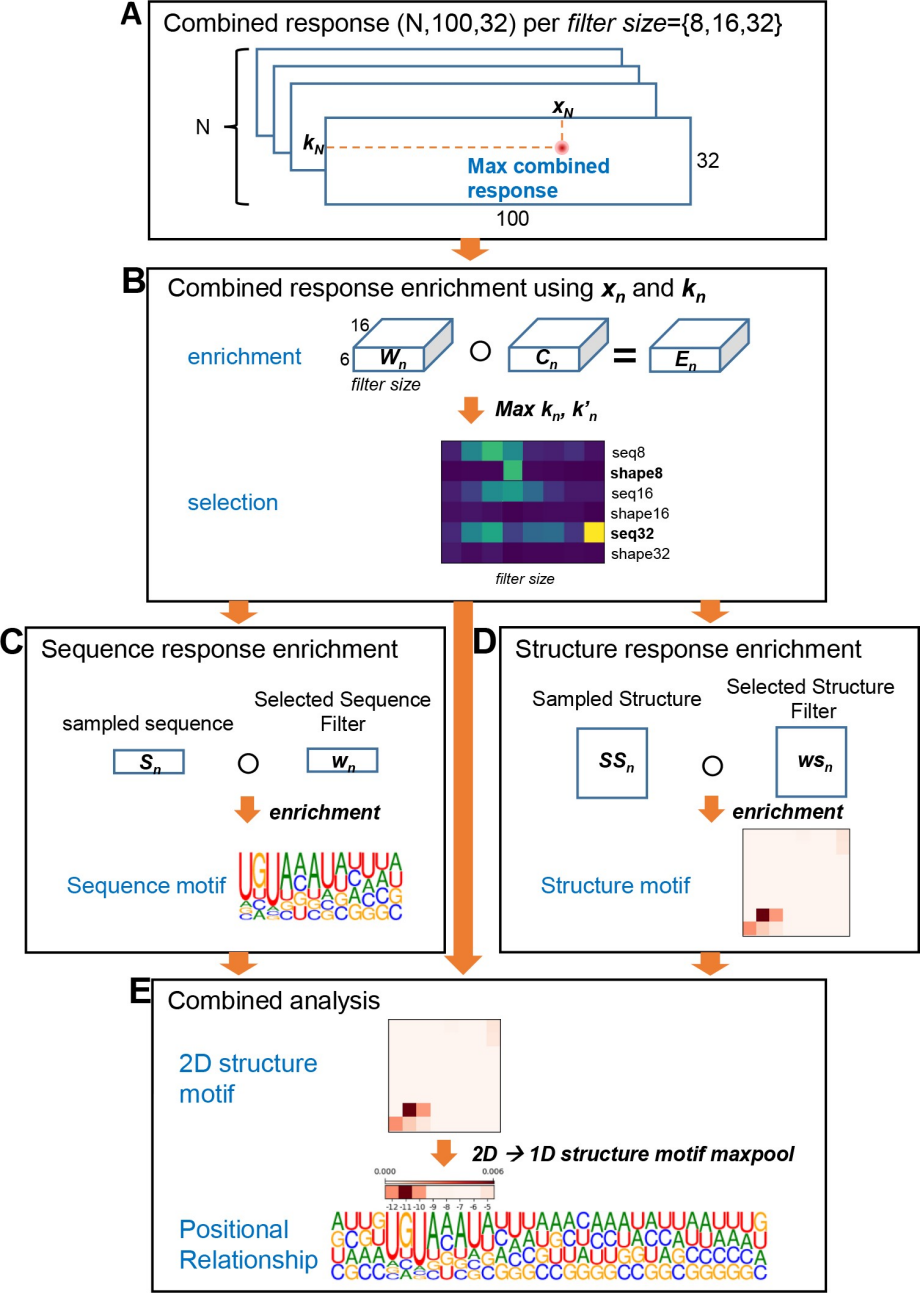
Motif extraction using response enrichment. For this example, we show motif extraction of PUM2. (A) Combined response of positive sample N was analyzed to retrieve maximum response resulting ***x***_***n***_ and ***k***_***n***_, where ***x*** is sequence position and ***k*** is filter index number. (B) Combined response enrichment was performed by element-wise product between weight *W* of selected filter ***k*** and sampled combined representation ***C***. By calculating response score of this enrichment, important filter types for PUM2 was selected. In this example shape8 and squence32 was selected. (C) By using information from previous steps, sequence response enrichment was performed for sequence32. Similar to combined response enrichment, sampled sequence information and selected sequence filter weight w was enriched. Enriched sequence response was transformed into PWM using softmax function and WebLogo was used to draw LOGO representations. (D) Structure enrichment was calculated in a similar way as sequence enrichment. Since filter used for structure convolution was 2-dimensional matrix, enriched structure was also 2-dimensional matrix. (E) For combined analysis, sequence motif and structure motif enriched from previous steps were aligned using relative enrichment position derived from combined response enrichment in (B). For simplicity, 2-dimensional structure motif was transformed into 1-dimensional array using max pooling operation.

In previous works, sequence motif enrichments were performed by aligning and scoring positive sample data by using the trained models. For example, in DeepBind, RBP binding sequences were fed into the trained model to calculate the scores, and then the maximum scoring positions were chosen and used to align input sequences. After sequence alignments, position weighted matrices (PWM) were calculated for each target and then converted into logo representations [8] using WebLogo [22]. In this work, we developed a new filter response enrichment method to extract and locate sequence, structure, and combined motifs. We name this method filter response enrichment since the procedure is similar to motif enrichment method proposed by DeepBind. The main difference is data being enriched; in our method weighted filter responses were enriched rather than aligned sequences. From this point and throughout this article, we will state this method as response enrichment for simplicity purpose. One of the advantages using deep CNN is that convolution filters are trained to mimic prevalent patterns of target data, in our case, RBP binding sequence and structural motifs of RNA. Therefore, by applying appropriate scoring schemes on trained filters, sequence and structural motifs can be extracted by enrichment analyses of selected filters. Our new motif extraction method for RBP PUM2 as an example is summarized in (Fig 4).

Using newly developed motif-finding method, we extracted sequence and structure motifs discovered by mmCNN. In order to compare and verify our findings, we compared our motifs with those of two prediction methods (DBN+ [9] and GraphProt [7]) and two literature evidences (RBPgroup [23] and CISBP-RNA [24]). RBPgroup collected 84 CLIP-seq of 48 human RBPs and applied non-negative matrix factorization on occupancy profile matrix to cluster RBPs. RBP binding motifs calculated for each RBP group were statistically more significant than individual RBP binding motif. CISBP-RNA database was created by experimental method RNAcompete, where 240,000 short (30~41 nucleotides) were generated and binding affinity with 207 different RBPs were measured.

**Fig 5:**
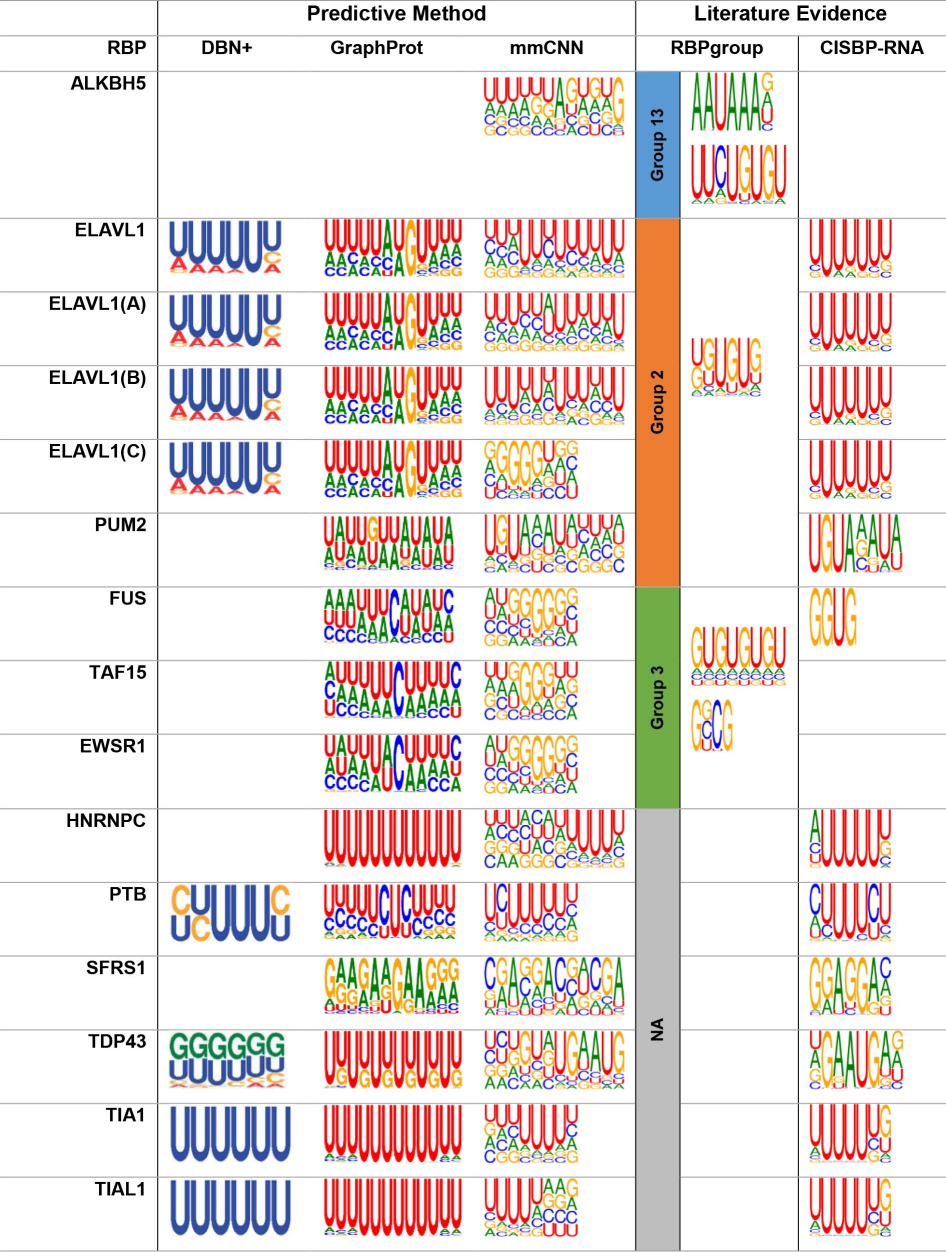
Sequence motif found by mmCNN and comparison with various researches. mmCNN was compared with two predictive method (DBN+ and GraphProt) and two other literature evidences (RBPgroup and CISBP-RNA). Since RBPgroup clustered different RBP families and found de novo motifs, individual groups found by RBPgroup was labeled in separate column.

We found that sequence motifs found by our method were more consistent with the evidences than other predictive methods (Fig 5), for sequence motif extraction using previous researches see (S4 Fig). The statistical significance of motifs was calculated using AME MEME-suite version 5.0.5 [25]. (i) ALKBH5 is known to be involved in polyadenylation [26,27] and AAUAAA consensus motif is known as a polyadenylation signal (P = 6.11e^-2^). While other two methods failed to find any meaningful motif, our method succeeded in finding sequence motif that well matched with this result. In addition, GU-repeat motif suggest by RBPgroup could be also observed. (ii) Since ELAVL1 family have 4 different CLIP-seq datasets, we searched for the sequence motifs for all 4 datasets separately. Previously, ELAVL family was found to have AU-rich binding motif by various researchers [7,9,28], but de novo motif found by RBPgroup was UGUGUG motif. Interestingly, ELAVL1 sequence motif found by our method have UG-rich motif or U-rich motif, which agrees with both RBPgroup and CISBP-RNA (where P-value of ELAVL1, ELAVL1(A), ELAVL1(B), and ELAVL1(C) were P = 2.15e^-34^, P = 6.64e^-36^, P = 2.48e^-40^, and P = 9.90e^-1^ respectively). The difference between sequence motifs of ELAVL1 is due to the different response enrichment positions, which will be discussed in the next section. (iii) Sequence motif for PUM2 was specified as UGUAAAUA according to CISBP-RNA, and RBPgroup suggested UG-rich motif. Enriched sequence was very similar to CISBP-RNA, but when enrichment window was wider, UG-rich regions were also observed (P = 2.29e^-1^). (iv) While FUS is known to have AU-rich loop structure [29], GU-repeat motif and GGUG motifs were newly found by RBPgroup CISBP-RNA, respectively. In agreement with these results, we could locate GU-rich regions of FUS. Interestingly, FUS, TAF15, and EWSR1 were known to have similar binding sequence motif by various literatures, our method predicted similar sequence motif for these RBPs, which was GU-rich region (where P-value of FUS, TAF15, and EWSR1 were P = 3.11E^-1^, P = 8.72E^-1^, and P = 3.62E^-1^ respectively). (v) For HNRNPC, AUUUUU motif by CISBP-RNA was more consistent with our motif than U-rich motif found by GraphProt (P = 1.23e^-36^). (vi) PTB is known to have CUUUUC binding motif [30], which was also found by CISBP-RNA. We found similar binding motif for PTB, which was CUUUU/C (P = 2.38e^-48^). (vii) Many previous researches suggested GA-repeat as sequence motif for SFRS1. Similar to this sequence, we found GAGGAC motif for SFRS1 (P = 8.73e^-17^), which had close resemblance with CISBP-RNA motif. (viii) For TDP43, which has TARDBP as alias, various researches suggested different binding motifs. For example, UG-repeat was suggested as sequence motif for TDP43 [29,31], and CISBP-RNA found UGAAUGAG. Interestingly, our model detected combinations of these two RBP binding motifs for TDP43 (P = 2.79e^-52^). (ix) It is well known TIA1/TIAL1 family prefers U-rich motif with various size [32], we found U-rich motif with length 5, which had high resemblance with CISBP-RNA (for TIA1 P = 1.95e^-26^ and TIAL1 P = 2.53e^-17^). Even though some RBP motifs found had low statistical significance, FET family (FUS, TAF15, and EWSR1) had similar motifs and newly found PUM2 motif had strong resemblance with CISBP-RNA motif.

### Combined response enrichment analysis captures two different RBP binding sequence motifs of ELAVL1 family

**Fig 6:**
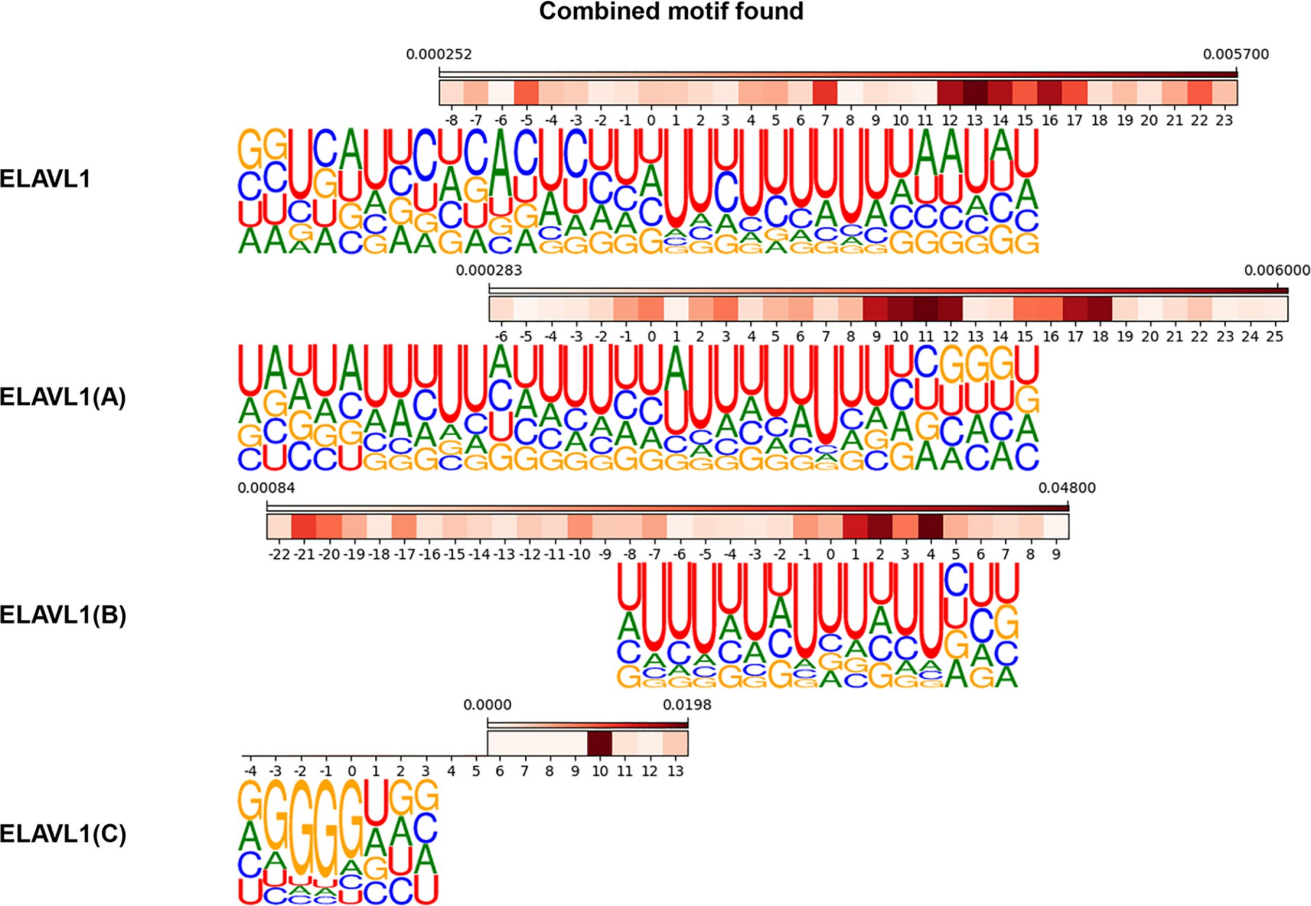
Combined motif analysis of ELAVL1 family. Aligned sequence motifs and structure motifs were shown for four different ELAVL1 family. Since we used weighted sum score for structure motif, values indicated in color bars are not structure forming probabilities but weighted scores. As the score is higher, a nucleotide is likely to participate in secondary structure.

In the previous section, we mentioned that two different motifs (UG-rich motif and U-rich motif) were found by other researches and both motifs were found by mmCNN in different ELAVL1 datasets. When analyzing positional relationship of sequence and structure motif for each ELAVL1 dataset, we found that difference between the two sequence motifs could be distinguished by the presence of secondary structure around the sequence motif site (Fig 6). For example, ELAVL1, ELAVL1(A), and ELAVL1(B) have a high structure score around C or A residue surrounding U-rich region (for ELAVL1 at position -5, 7 and 12~17, for ELAVL1(A) around position -1~0, 2~3, and 9~12, and for ELAVL1(B) at position 1~4). In these RBPs, U-rich regions were present in the hairpin-loop region, which was maintained by the stem forming positions. By contrast, for ELAVL1(C), secondary structure was indirectly related with sequence motif region, where strong stem forming position was detected at position 10. Interestingly, U-rich motif and de novo GU-rich motif seems to have preference for a single stranded RNA (ssRNA), which agrees with other research [28].

### Longer sequence motif detector captures de novo motif for ALKBH5 and IGF2BP123

**Fig 7:**
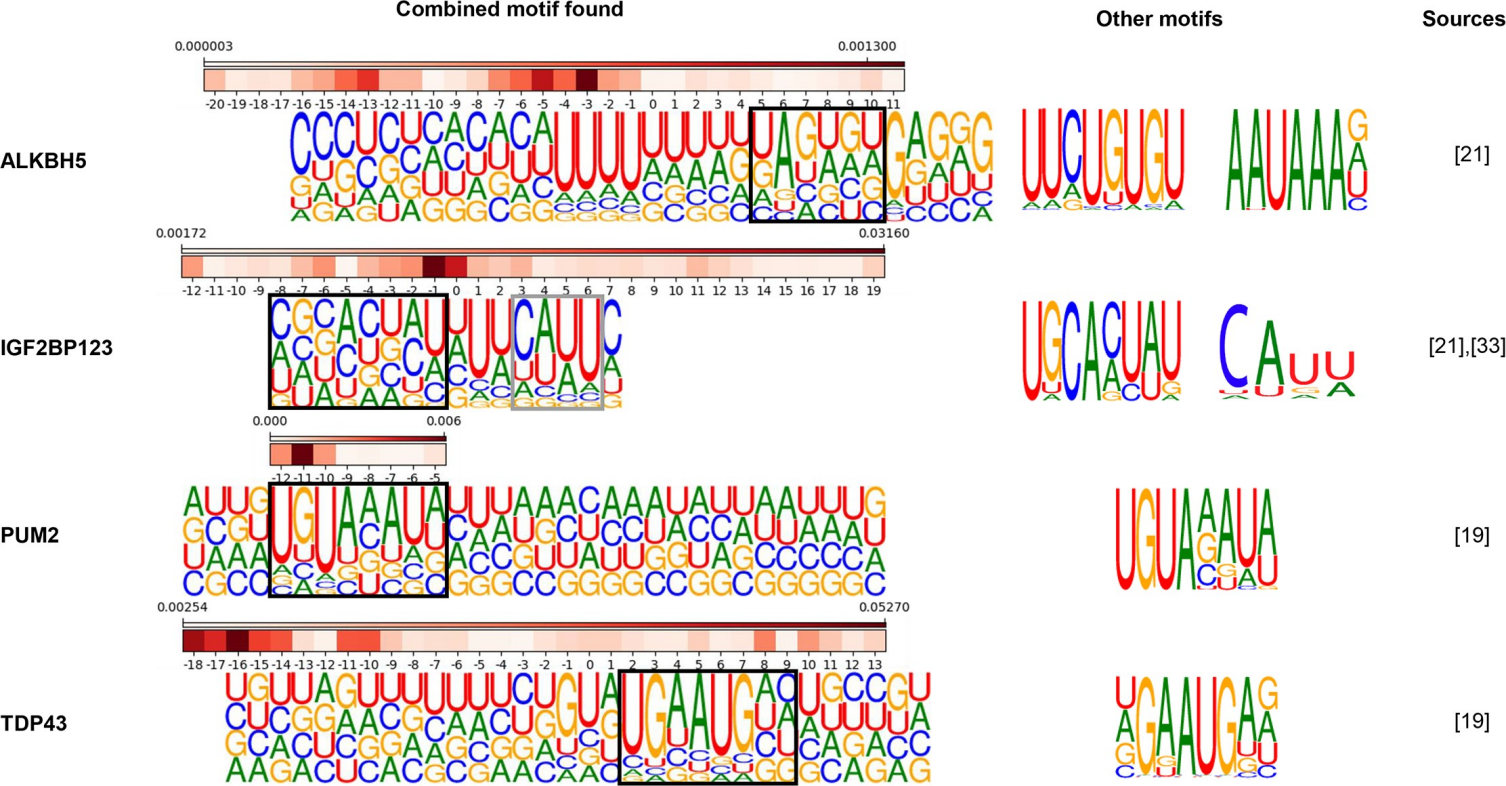
Combined motif analysis of other RBP families. Full-sized sequence motif enriched by response enrichments were aligned with structure motifs. Sequence motifs found by other researches were indicated in separate column for comparison and matching sequence motifs were indicated with black or grey boxes.

It was found that the prediction accuracy was greatly improved for ALKBH5 when structure information was included. We reasoned that structure probability matrix that we introduced in this work was suitable for detecting ALKBH5 structural motif. From various researches [33,34], ALKBH5 is known to have a function of demethylation of N^6^-Methyladenosine (m^6^A). According to these studies, the homology modeling structure of ALKBH5 has binding preference to ssRNA since it has amino-acid residues that interrupt binding to the double stranded RNA (dsRNA). We found that sequence motif predicted by mmCNN was U-rich region surrounded by hairpin-loop structure in the upstream region and de novo motifs AAUAAA and GUGU were found in the downstream region, see (Figs 5 and 7). These motifs found to have lack of structures, i.e. loop region or ssRNA. However, hairpin-loop region present upstream of sequence motif was also predicted to be important. Therefore, we suggest that secondary structure in the upstream region might guide ALKBH5 to bind de novo sequence motif, which prefers ssRNA. For another example, binding regions of IGF2BP123 seems to have similar characteristics. Sequence motif of IGF2BP123 known to contain distinct tri-ribonucleic acid CAU [35], and motif found by mmCNN was CAUU surrounded by U-rich region (P = 1.23e^-4^). Interestingly, when full-sized sequence motif (*filter size* 16) was aligned with structure motif, de novo motif proposed by RBPgroup GCACUAU could be observed in the upstream region of CAUU motif. It seems that de novo motif prefers stem structure (at position -7~3) and CAUU seems to prefer hairpin-loop structure (at position 3~11), which agrees with structure motif proposed by GraphProt.

### Combined effect of Sequence and structural features improves sequence motif search: PUM2 and TDP43

By using sequence and structure features together, we found better sequence motifs for PUM2 and TDP43 (Fig 7). According to previous research [36], PUM2 has preference for hairpin structure, which agrees with our result that the probability of having hairpin-loop was higher at the RBP binding site. In our result, RBP binding sequence motif UGUAAAUAU and hairpin-loop structure motif were co-occurring in the same region. For TDP43, which has alternative name TARDBP, our model predicted sequence motif similar to UGAAUGAG, which resembles motif found by RNAcompete experiment. Similar to PUM2, sequence motif found for TDP43 was located in the hairpin-loop region.

## Conclusion

In this work, we developed a bimodal multi-sized filter deep convolution neural network using sequence and secondary structure information to detect various sized primary, secondary and combined motifs. Many previous works have tried to predict RBP binding sites on RNA using various computational methods with various RNA sequence-derived information [7–9,37]. In this work, we have focused on developing a new deep CNN architecture that integrates sequence and structure information to achieve higher prediction accuracy by accurately locating the RBP binding motif sites by exploiting synergistic effect of sequence and structure of RNA molecules. We showed that we were able to achieve higher prediction accuracy by using multiple filters of different sizes, compared to single-sized filter method. We also introduced a new type of RNA structure feature by gathering multiple secondary structures derived from RNAshapes to create the structure probability matrix. By using the structure probability matrix, we achieved roughly 4% improvement. In some cases, performance gain was much higher. For example, in the case of ALKBH5, relative error reduction rate was improved by 30%.

By introducing response enrichment method, we could extract high quality RBP binding sequence motifs, which had high resemblance with sequence motifs discovered by RBPgroup and CISBP-RNA. Among 24 RBPs, sequence motifs for ALKBH5, AGO2, ELAVL1 family, FUS, HNRNPC, PUM2, PTB, TDP43, TIA1, and TIAL1 could be extracted successfully. Especially, predicted motifs for ALKBH5, PUM2, and TDP43 had distinct sequence motif where no other prediction method predicted correctly.

By integrating positional information to a deep learning architecture, we could analyze positional relationship between sequence and structure motifs. For some RBP families, sequence motif and structural motif were closely interrelated. For example, sequence motif of PUM2 and TDP43 had distinct hairpin loop structure at sequence motif region. For ALKBH5 and IGF2BP123 had strong hairpin loop presence in the upstream region of RBP binding site. There was no reference referring to importance of hairpin loop structure in ALKBH5, but our result and other prediction methods suggested importance of the structure information for ALKBH5 binding site prediction.

## Methods

### Data preparation

For this work, we used CLIP-SEQ dataset that we downloaded from http://www.bioinf.uni-freiburg.de/Software/GraphProt/. We used this dataset since it is well-balanced dataset and constructed for RNA structure calculation. It is known that at least 150 nucleotides are needed to calculate good RNA secondary structures [38]. Therefore, we trimmed the original RNA sequences so that the length of all RNA sequences became 200, with RBP binding sites placed in the middle of the sequences. We converted these sequences into one-hot encoded representations as in DeepBind [8]. Since the maximum sequence length of our RNA data was set to 200, one-hot encoded sequences had the dimension of (200, 4).

Structure information was calculated from RNA sequences using RNAshapes [21]. For a given RNA sequence, this program produces multiple secondary structures which are sorted by their free energies in kcal·mol^-1^ [21]. From these structures, we used top 100 structures to convert those energies to probabilities using Boltzmann distribution formula;

$$p_{l}=\frac{e^{-\frac{\varepsilon_{l}}{kT}}}{\sum_{m=1}^{M}e^{-\frac{\varepsilon_{m}}{kT}}},$$ (1)

where *ε*_*l*_ is the energy of the structure *l*, *k* is Boltzmann constant (= 0.001987 kcal·mol^-1^·K^-1^), and *T* is temperature (= 310.15 K). We neglected secondary structures beyond the top 100 structures since probability contributions of those structures were negligible. Additionally, we compared performance of mmCNN using top 100 and top 1, top 100 performed better, see (S5 Fig). Next, using the top 100 structures and their probabilities, we calculated the probability for a pair of residues, *i* and *j*, forming stem secondary structure, *p*(*i*, *j*), using the following formula,

$$p(i,j)=\sum_{l=1}^{100}p_{l}(i,j),$$ (2)

where *p*_*l*_(*i*,*j*) is either *p*_*l*_ if a pair of residues, *i* and *j*, is interacting in structure *l*, or 0 otherwise. Other secondary structures such as inter-loop, bulge-loop, hairpin-loop, multi-loop, and external regions can be expressed using stem structures, for details see (S6 Fig). Since the maximum sequence length of our RNA data was set to 200, structure input matrices had the dimension of (200, 200).

### Designing of multi-modal multi-sized filter deep convolutional neural network

Since CLIP-seq dataset contains various length of RBP binding sites ranging from 25 to 75, we developed a module named “multi-sized convolution module” that was designed to capture sequence and structure motifs with different sizes. The module consists of three 2-dimensional convolution filters with dimension of (8, *x*), (16, *x*), and (32, *x*) where *x* is the size of “height”, followed by ReLU activation layer and max pooling layer, for sequence convolution height was 4 and for structure convolution height for each filter was 8, 16, and 32. For combined convolution, height was equal to the number of stacked convolution output from the previous layer. Three separate outputs from max pooling layers are stacked together to produce a single final output (Fig 2A). Weights of each convolution layer were initialized using Xavier uniform (Glorot uniform) initializers and biases were initialized to zero. For motif extraction layer, 16 filters were used for single convolution [39].

Since convolution layer on raw data act as a motif detector, the convolution output of this layer represents motif matching score on 200 residue positions. In order to extract combined features from sequence and structure inputs, we processed sequence and structure inputs with separate convolution layers and concatenated these outputs into combined representation. We used three different sized filters for sequence and structure, concatenation function was used to combine 3 outputs from sequence and 3 outputs from structure convolution output. To consider the complexity of RBP binding motif consisting of sequence and structural motifs, we introduced three additional multi-sized convolutional modules on concatenated output from the previous layer. We used three filters with the size of 8, 16, and 32. Weights and bias were initialized as same as motif extraction convolution layer. For combined motif extraction layer, 32 filters were used for single convolution. Since complex motif can be a combination of distant simple motifs, we continuously reduced our data size into half to eliminate non-matching positions by using max pooling layers. To avoid overtraining, we used dropout layers with keep-probability of 0.75 after sequence convolutional layer and structure convolutional layer, and a dropout layer with keep-probability of 0.5 right before fully connected layer. For optimizer, we used Adadelta with default parameters, *learning rate* = 1.0, *rho* = 0.95, *epsilon* = *None*, and *decay* = 0.0, which was recommended not to be changed according to Keras manual, see for more information about optimizer selection in (S1 and S2 Tables and S7 Fig).

### Tenfold cross validation and ensemble model

There are two separate CLIP-SEQ sequence data in GraphProt, which are labeled as *train* and *ls*. We used *train* set for training and validation of the model, and *ls* as an external test set for final AUC calculation. We divided the training dataset into ten segments of equal size and performed 10-fold cross validation to select the optimal model parameters. The final ensemble model was derived by averaging the output scores from these ten models, and then the final performance evaluation was made on the external test dataset.

### Motif extraction using response enrichment

In this work, we developed a new response enrichment method to extract and locate sequence, structure, and combined motifs. Motif extraction method for RBP PUM2 as an example is illustrated in (Fig 4). The positions of the response extraction points are depicted in (Fig 2). The new motif extraction methods proceed as following steps.

#### Combined response analysis

Combined responses were calculated by feeding *N* positive samples of a RBP to the trained mmCNN model (Fig 4A). For each positive data *n* (where *n* is an index of positive samples, *n* ∈ {1,…,*N*}), combined response matrix having size of (100, 32) was analyzed to find a position *x*_*n*_ and a combined filter index *k*_*n*_ which produces the maximum combined response (where *k*_*n*_ ∈ {1,…,32} since 32 filters were used, and x_n_ ∈ {1,…,100} due to the max pooling in the previous convolution step). Since we used three filters with different sizes for each convolution, combined response analysis was done for each filter size (where *filter size* ∈ {8,16,32}).

#### Combined response enrichments

Combined response enrichments were calculated using *x*_*n*_ and *k*_*n*_ from the previous step (Fig 4B). Here, we first define a sampled combined representation, *C*_*n*,*x*,*t*,*k’*_, where *n* represents a positive sample index, *x* is position, *t* is filter type of previous convolution (*seq8*, *shape8*, *seq16*, *shape16*, *seq32*, *shape32*), and *k*’ is filter index of previous convolution (sequence or structure convolution step, where *k*’ ∈ {1,…,16}). We used *x*_*n*_ as a center of extraction point to sample $C_{X_{n},t,k^{\prime }}$ for each positive sample *n*. Since *x*_*n*_ is the center of extraction point, sampled combined representation can be defined as

$$C_{X_{n},t,k\prime }=\left\{ \begin{array}{c} x_{n}-\frac{filter\;size}{2}\leq X_{n}<x_{n}+\frac{filter\;size}{2}\\ 0\leq t<6,(seq8,shape8,seq16,shape16,seq32,shape32)\\ 0\leq k^{\prime }<16 \end{array}.\right.$$ (3)

Therefore, the dimensionality of $C_{X_{n},t,k^{\prime }}$ is (*filter size*, 6, 16). By using max *k*_*n*_ operation from previous step and combined convolution filter *W*, combined response enrichment can be defined as element-wise product of sampled combined response and the combined convolution filter. Then, the combined response enrichment of *N* samples can be defined as

$${\left[combined\;response\;enrichme{nt}_{filter\;size}\right]}_{X,t}=\underset{k\prime }{\max}\left[\sum_{n}^{N}C_{X_{n},t,k^{\prime }}○W_{X,t,k\prime ,\max k_{n}}\right],$$ (4)

where ○ represents element-wise product of two 2-dimensional matrices. Since response enrichment represents relative importance of positions of sequence and structure motif detector, to eliminate noise and extract clear motif we performed $\max k_{n}^{\prime }$ operation.

#### Selection of best enrichment using response score

In order to select the best enrichment, response scores for each filter types were calculated (Fig 4B). Since different filter size may cause different enrichment size, direct comparison between these enrichments were difficult. In order to compare enrichments with different sizes, we eliminated *x* by using max operation along this dimension,

$${\left[response\;score\right]}_{filter\;size}=\sum_{t}\underset{X}{\max}\left[{\left[combined\;reseponse\;enrichme{nt}_{filter\;size}\right]}_{X,t}\right].$$ (5)

For an example case (Fig 4B), *filter size* = 8 was selected by comparing response score of three enrichments from three different filter sizes. By comparing response scores, sequence filter with size 32 (*t = seq32 or in matrix index number t = 5*) and structure filter with size 16 (*t = shape16 or in matrix index number t = 3*) have maximum enrichment scores among other convolution types. Considering $\frac{filter\;size}{2}$ as enrichment center (due to padding in convolution operation), we calculated difference *d*_*t*_ between center position and max-enriched position as shifting constant for sequence and structure enrichment (Fig 4B).

#### Sequence response enrichments

For sequence response enrichment, the same data position *x*_*n*_ was used to sample one-hot encoded sequence data in a similar way (Fig 4C). Since *x*_*n*_ is derived from maxpooled output, we considered max pooling effect and shifting constant *d*_*t*_ by selecting position having higher response values at position 2(*x*_*n*_ + *d*_*t*_) and 2(*x*_*n*_ + *d*_*t*_) + 1 of sequence responses. Sampled sequence $S_{X_{n},y}$ can be defined as,

$$S_{X_{n},y}=\left\{ \begin{array}{c} {P(x}_{n},d_{t})-\frac{filter\;size}{2}\leq X_{n}<{P(x}_{n},d_{t})+\frac{filter\;size}{2}\\ 0\leq y<4,(A,U,G,C) \end{array},\right.$$ (6)

where *P*(*x*_*n*_,*d*_*t*_) is either 2(*x*_*n*_ + *d*_*t*_) and 2(*x*_*n*_ + *d*_*t*_) + 1, position with higher sequence response was selected. For example case in (Fig 4C) *filter size* = 32 and *t* = 5. Sequence response enrichments were calculated similar as combined response enrichments, which can be calculated by elementwise product between sampled sequence and selected sequence filter $w_{\max k_{n}^{\prime }}$. Sequence response enrichment can be defined as

$${\left[sequence\;response\;enrichment_{filter\;size}\right]}_{X,y}=\sum_{n}^{N}W_{z,t,\max k_{n}^{\prime },\max k_{n}}S_{X_{n},y}○w_{X,y,\max k_{n}^{\prime }},$$ (7)

where $W_{z,t,k_{n}}$ is weighting factor from combined filter which varies due to positive sample index, *z* is maximum enrichment position of selected convolution type *t* calculated from previous step,

$$z=\underset{X}{\arg\;\max}\left[{\left[combined\;response\;enrichme{nt}_{filter\;size}\right]}_{X,t}\right],$$ (8)

where $\max k_{n}^{\prime }$ represents sequence convolution filter index which has maximum sequence response value at position 2(*x*_*n*_ + *d*_*t*_), max *k*_*n*_ is combined filter index which result maximum combined response value at same position, and 0 ≤ *X* < *filter size*. Since sequence or structure information smaller than *filter size* might contain noise, for sequence and structure response enrichments in boundary was neglected.

#### Structure response enrichments

For structure response enrichment, the same procedure as sequence response enrichment was performed, for the example *filter size* = 8 and *t* = 1 see (Fig 4D). Since structure information is 2-dimensional information, we sampled pairwise structure forming probability as similar to sequence response enrichment. Sampled structure ${SS}_{X_{n},Y_{n}}$ can be defined as

$${SS}_{X_{n},Y_{n}}=\left\{ \begin{array}{c} {P(x}_{n},d_{t})-\frac{filter\;size}{2}\leq X_{n}<{P(x}_{n},d_{t})+\frac{filter\;size}{2}\\ Y_{n}=X_{n} \end{array}.\right.$$ (9)

For example case, *filter size* = 8 and *t* = 1, see (Fig 4D). When extracting structure response, see (Fig 2), max pooling operation decreases structure convolution output into (100, 1) dimension. To consider this effect and we noticed that structure probability matrix had strong signal in local regions, we used the same extraction range for *Y*_*n*_ as *X*_*n*_ when extracting structure samples. Therefore, structure response enrichments can be defined as

$${\left[structure\;response\;enrichment_{filter\;size}\right]}_{X,Y}=\sum_{n}^{N}W_{z,t,\max k_{n}^{\prime },\max k_{n}}{SS}_{X_{n},Y_{n}}○ws_{X,Y,\max k_{n}^{\prime }},$$ (10)

where *ws* is structure filter weight, $\max k_{n}^{\prime }$ is structure filter index which has maximum structure response value at position *P*(*x*_*n*_,*d*_*t*_), 0 ≤ *X* < *filter size*, and 0 ≤ *Y* < *filter size*.

### Combined analysis

For combined analysis, we used (Equation *combined response enrichment*) to find positional relationship between sequence and structure motif, see (Fig 4B). Positional relationship could be calculated by comparing *d*_*t*_ of selected sequence and structure motif. For simplification and major structure information retrieved by our method was structure forming probability in local region, we transformed extracted 2-dimensional structure motif into 1-dimensional array, which can be seen in (Fig 4E). Then extracted sequence motif and structure motif were aligned using *d*_*t*_ and compared with various literature evidences.

### Sequence response enrichments to sequence LOGO representations

Since *sequence response enrichments* were weighted nucleic acid propensities at specific position, we applied softmax function on these enrichments to convert weighted propensities into probability matrix. For some RBPs, long sequence motif detectors were preferred. For clarity, we selected maximum scoring 12-sized positions for these RBP families using sum scores resulted from scanning with 12-sized window, as in (Fig 4). Then, sequence motif was expressed into sequence logo using WebLogo [22].

## Supporting information

S1 Fig Comparison between structure using mmCNN and non-structure mmCNN. Only small number of RBP improved when structure information was used. ALKBH5, PUM2, and QKI showed improved performance (relative error reduction rate improved by ALKBH5 3.4%, PUM2 23%, and QKI 25%)
(PDF)

S2 Fig Relative error reduction (RER) of RBP-24 between both information using mmCNN vs sequence information using mmCNN. Various network types with different number of convolution layers were used (L1, L2, L3, and mmCNN) to see diminishing effect of structure information due to stacking more convolution layers.
(PDF)

S3 Fig Boxplot of RBP-24 AUCs and RERs of four different network types, L1, L2, L3, and mmCNN. Interestingly, mmCNN, which contain 4 layers of multi-sized filter convolution module, seems to have the best performance AUC. Network type L2 seems to have best structural feature usage, which is measured by RER see (S4 Fig, and structural feature usage decreases as the more convolution layers were stacked.
(PDF)

S4 Fig Sequence motif calculated using max sequence response score as alignment center. (PDF)

S5 Fig AUC comparison between secondary structure forming probability using top 100 secondary structure and single best secondary structure. Structure forming probability using top 100 was better than top 1.
(PDF)

S6 Fig Example of secondary structure and representation of RNA. (A) Secondary structure made using RNAfold provided by Vienna package. (B) Secondary structure representation of mmCNN, in main article only stem information was used since other secondary structures can be expressed using stem information only.
(PDF)

S7 Fig mmCNN model optimization using 7 sample RBPs. For optimizer selection 5 sets of weight initialization range and 5 running rates for Adadelta, Adam, Adagrad, and Rmsprop optimizers.
(PDF)

S1 Table Five different sets of convolution and dense weight initialization ranges. (PDF)

S2 Table Five different learning rates tested for four optimizers. (PDF)
