# Supplementary material for: Prediction of binding property of RNA-binding proteins using multi-sized filters and multi-modal deep convolutional neural network
Source: PLoS One. 2019 Apr 26;14(4):e0216257. doi: 10.1371/journal.pone.0216257 (PMC6485761; doi:10.1371/journal.pone.0216257)
Supplement: S1 Fig — Only small number of RBP improved when structure information was used. ALKBH5, PUM2, and QKI showed improved performance (relative error reduction rate improved by ALKBH5 3.4%, PUM2 23%, and QKI 25%) (PDF) [file pone.0216257.s001.pdf]

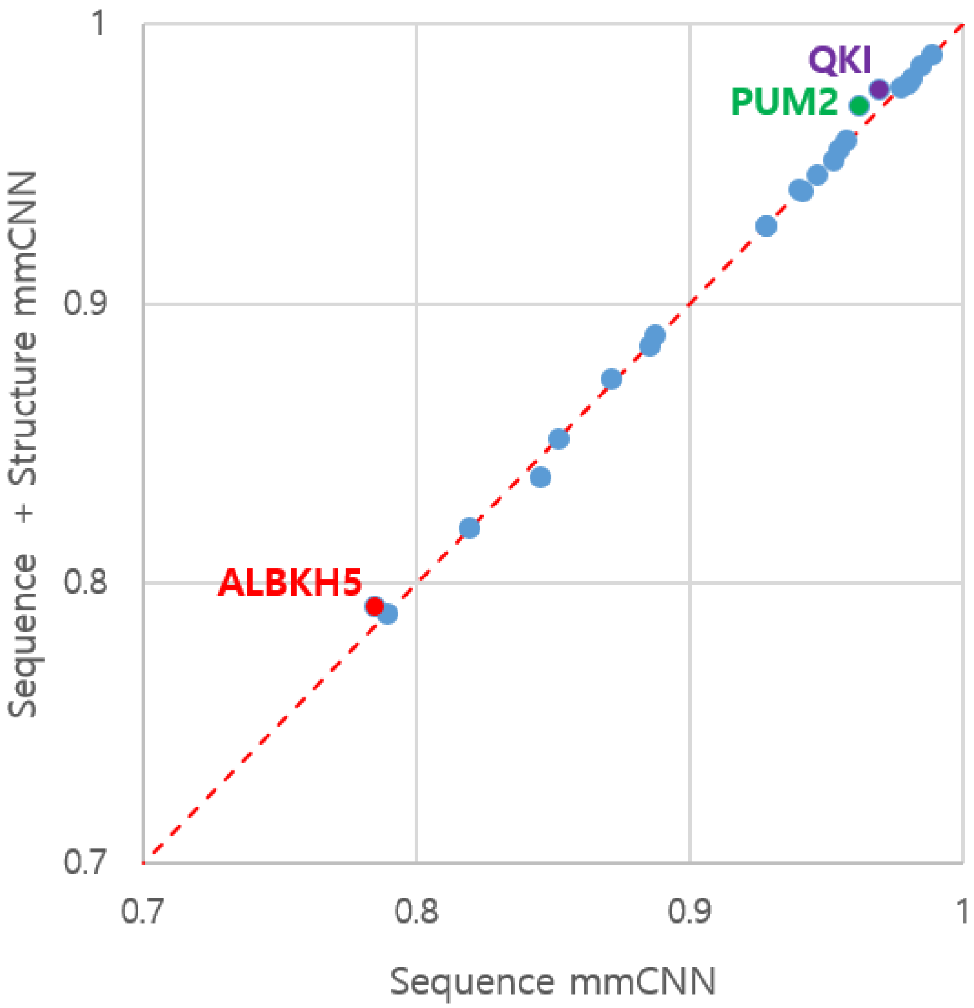

S1 Fig. Comparison between structure using mmCNN and non-structure mmCNN. Only small number of RBP improved when structure information was used. ALKBH5, PUM2, and QKI showed improved performance (relative error reduction rate improved by ALKBH5 3.4%, PUM2 23%, and QKI 25%)

In many researches, impact of secondary structure usage was calculated by comparing two prediction results with different feature usage, prediction power of sequence only deepCNN versus prediction power of sequence and structure feature using deepCNN, see (S3 Fig). We extracted two different prediction result by altering external test inputs for 24 RBPs. To calculate prediction results of mmCNN without structure information, we set structure input to zero-matrix to eliminate structure information being fed into mmCNN.
