# Supplementary material for: Prediction of binding property of RNA-binding proteins using multi-sized filters and multi-modal deep convolutional neural network
Source: PLoS One. 2019 Apr 26;14(4):e0216257. doi: 10.1371/journal.pone.0216257 (PMC6485761; doi:10.1371/journal.pone.0216257)
Supplement: S2 Fig — Various network types with different number of convolution layers were used (L1, L2, L3, and mmCNN) to see diminishing effect of structure information due to stacking more convolution layers. (PDF) [file pone.0216257.s002.pdf]

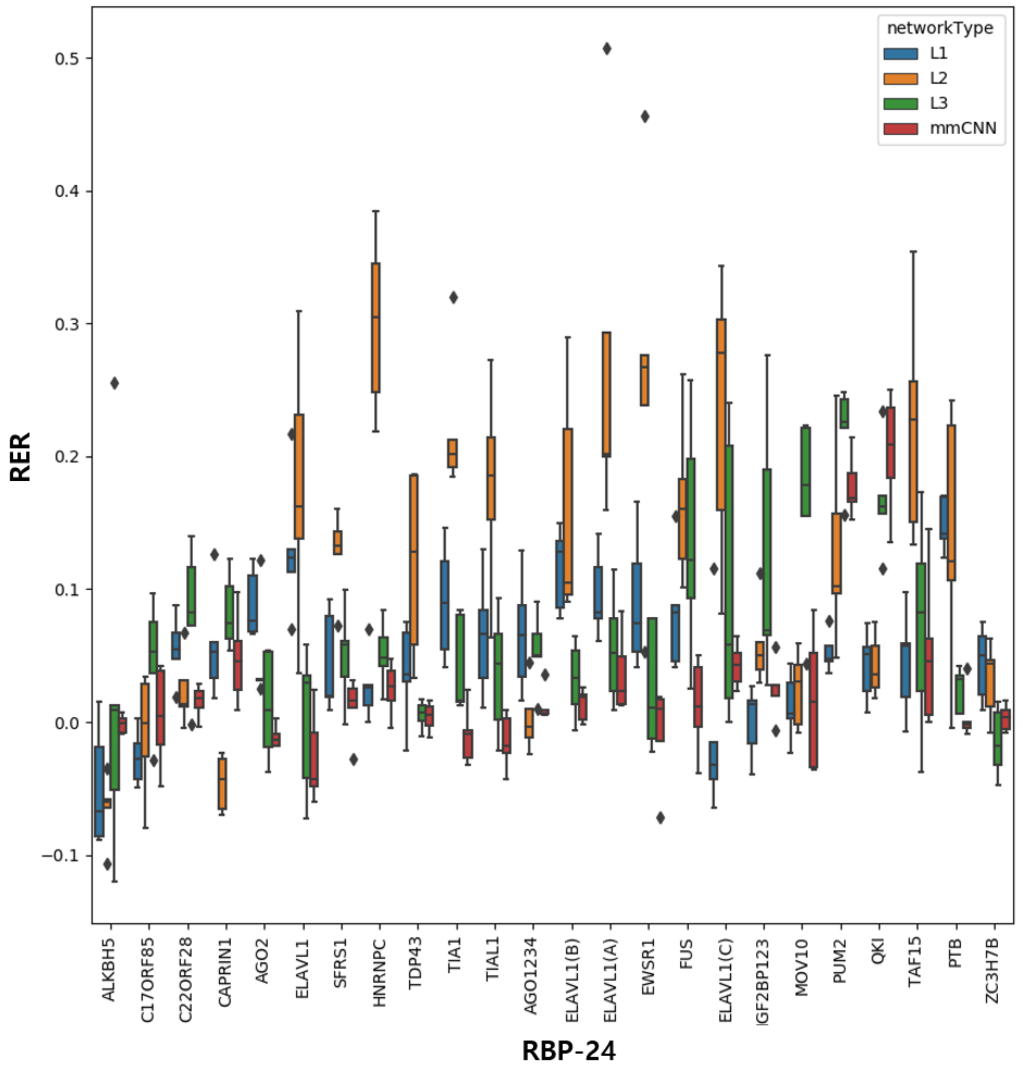

S2 Fig. Relative error reduction (RER) of RBP-24 between both information using mmCNN vs sequence information using mmCNN. Various network types with different number of convolution layers were used (L1, L2, L3, and mmCNN) to see diminishing effect of structure information due to stacking more convolution layers.

1. mmCNN: original model we used throughout the article, for more information see main article
2. L1: one-hot encoded sequence information and structure probability matrix followed by separate multi-kernel module, 64-node dense layer, and classification layer.
3. L2: Separate paths in L1 are combined using concatenation and followed by additional multi-kernel module, 64-node dense layer, and classification layer.
4. L3: Combined path in L2 followed by additional multi-kernel module, 64-node dense layer, and classification layer.
