# Supplementary material for: Prediction of binding property of RNA-binding proteins using multi-sized filters and multi-modal deep convolutional neural network
Source: PLoS One. 2019 Apr 26;14(4):e0216257. doi: 10.1371/journal.pone.0216257 (PMC6485761; doi:10.1371/journal.pone.0216257)
Supplement: S3 Fig — Interestingly, mmCNN, which contain 4 layers of multi-sized filter convolution module, seems to have the best performance AUC. Network type L2 seems to have best structural feature usage, which is measured by RER see (S4 Fig, and structural feature usage decreases as the more convolution layers were stacked. (PDF) [file pone.0216257.s003.pdf]

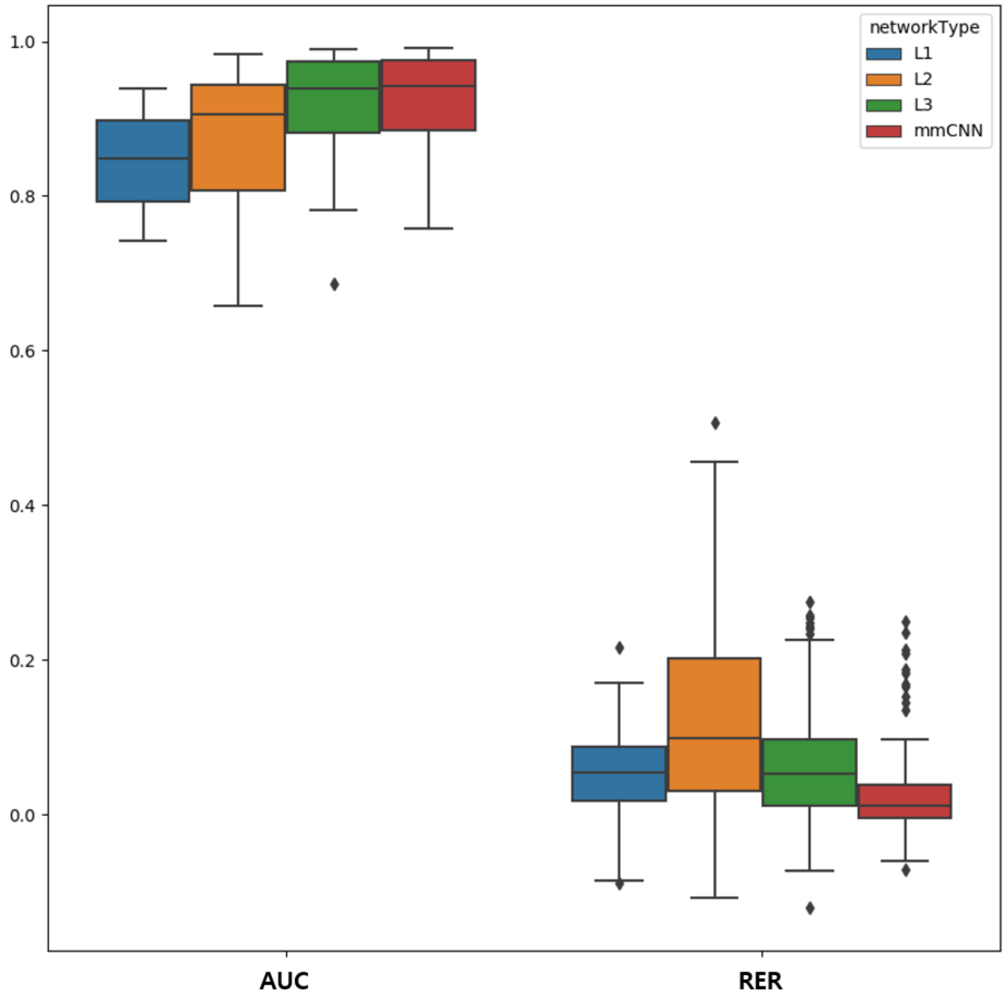

S3 Fig. Boxplot of RBP-24 AUCs and RERs of four different network types, L1, L2, L3, and mmCNN. Interestingly, mmCNN, which contain 4 layers of multi-sized filter convolution module, seems to have the best performance AUC. Network type L2 seems to have best structural feature usage, which is measured by RER see (Figure S4), and structural feature usage decreases as the more convolution layers were stacked.
