# Supplementary material for: Prediction of binding property of RNA-binding proteins using multi-sized filters and multi-modal deep convolutional neural network
Source: PLoS One. 2019 Apr 26;14(4):e0216257. doi: 10.1371/journal.pone.0216257 (PMC6485761; doi:10.1371/journal.pone.0216257)
Supplement: S4 Fig — (PDF) [file pone.0216257.s004.pdf]

| RBPs | Sequence Motifs | RBPs | Sequence Motifs |
|---|---|---|---|
| ALKBH5 | 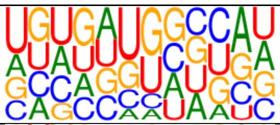 | ELAVL1(A) | 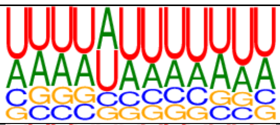 |
| C17ORF85 | 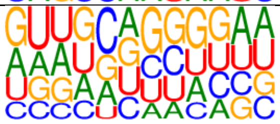 | ELAVL1(B) | 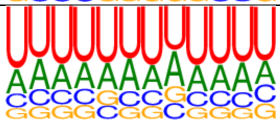 |
| C22ORF28 | 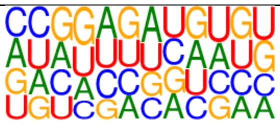 | EWSR1 | 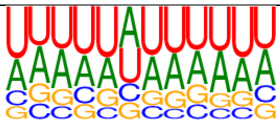 |
| CAPRIN1 | 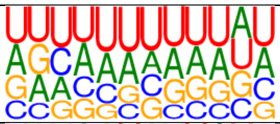 | FUS | 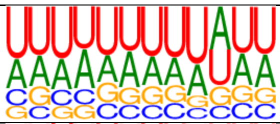 |
| AGO2 | 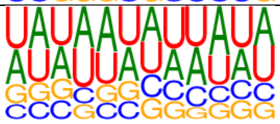 | ELAVL1(C) | 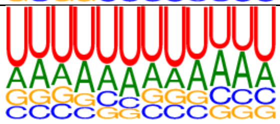 |
| ELAVL1 | 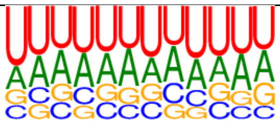 | IGF2BP123 | 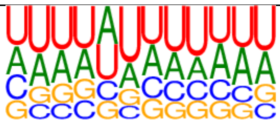 |
| SFRS1 | 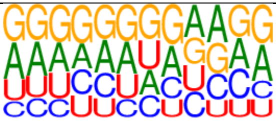 | MOV10 | 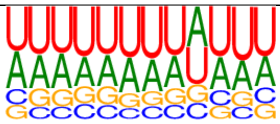 |
| HNRNPC | 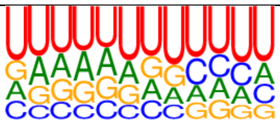 | PUM2 | 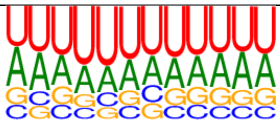 |
| TDP43 | 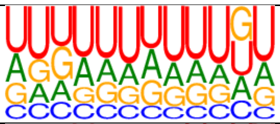 | QKI | 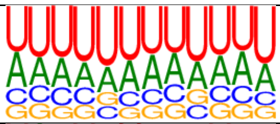 |
| TIA1 | 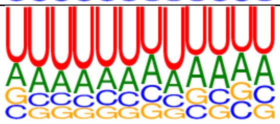 | TAF15 | 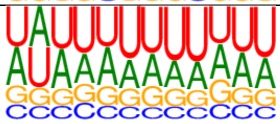 |
| TIAL1 | 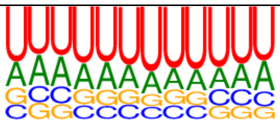 | PTB | 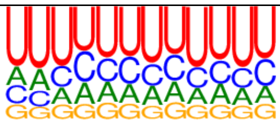 |
| AGO1234 | 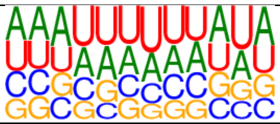 | ZC3H7B | 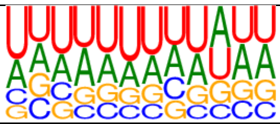 |

**S4 Fig. Sequence motif calculated using max sequence response score as alignment center.** For sequence motif calculation, we followed procedures used in DeepBind with only using maximum response sequence. We calculated response values of each positive sample data, which went through first convolution layer combined with rectification (sequence response extraction point in Fig 2). In contrast to DeepBind procedure where sequence positions having response greater than zero, we only aligned maximum response value per single positive sequence. We also aligned sequence positions non-zero positive values, but improvements sequence motif extraction was not significant.

After aligning sequences, position frequency matrix (PFM) was calculated and softmax function was applied to PFM. Sequence LOGO representations were calculated using this PFM with -D transfac -F png -a AUGC -A rna -U probability --composition equiprobable -X False -Y False -S 1 -s large --errorbars False --color green A 'A' --color orange G 'G' --color red U 'U' --color blue C 'C' option.
