# Supplementary material for: Prediction of binding property of RNA-binding proteins using multi-sized filters and multi-modal deep convolutional neural network
Source: PLoS One. 2019 Apr 26;14(4):e0216257. doi: 10.1371/journal.pone.0216257 (PMC6485761; doi:10.1371/journal.pone.0216257)
Supplement: S5 Fig — Structure forming probability using top 100 was better than top 1. (PDF) [file pone.0216257.s005.pdf]

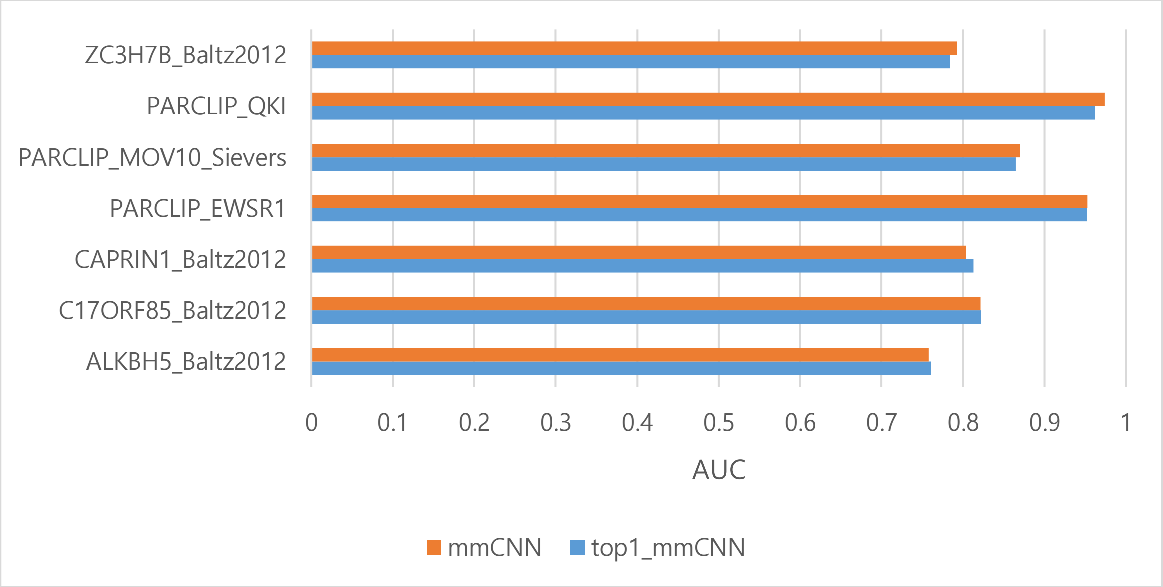

S5 Fig. AUC comparison between secondary structure forming probability using top 100 secondary structure and single best secondary structure. Structure forming probability using top 100 was better than top 1.
