# Supplementary material for: Prediction of binding property of RNA-binding proteins using multi-sized filters and multi-modal deep convolutional neural network
Source: PLoS One. 2019 Apr 26;14(4):e0216257. doi: 10.1371/journal.pone.0216257 (PMC6485761; doi:10.1371/journal.pone.0216257)
Supplement: S6 Fig — (A) Secondary structure made using RNAfold provided by Vienna package. (B) Secondary structure representation of mmCNN, in main article only stem information was used since other secondary structures can be expressed using stem information only. (PDF) [file pone.0216257.s006.pdf]

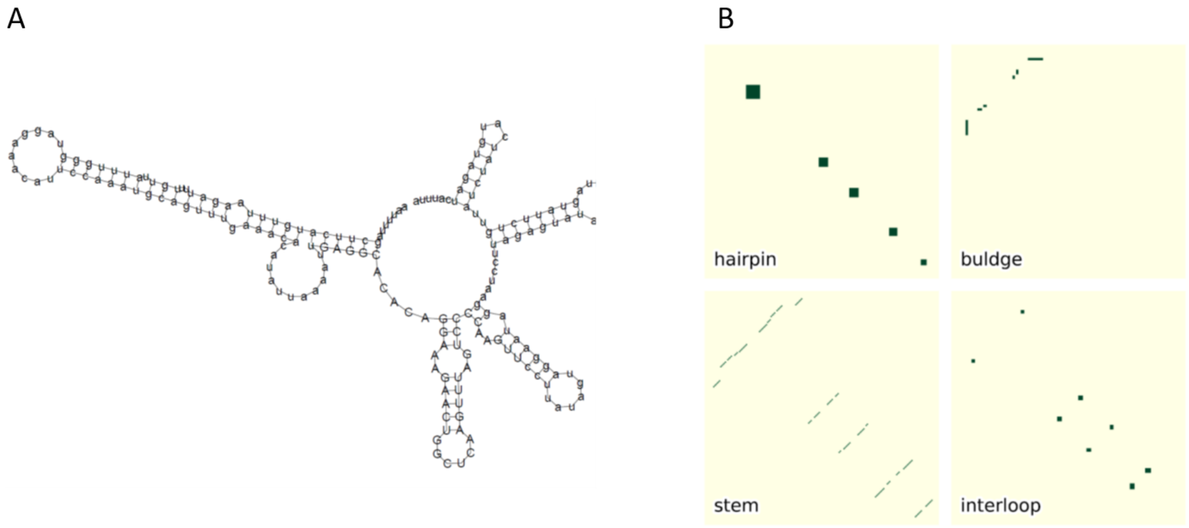

S6 Fig. Example of secondary structure and representation of RNA. (A) Secondary structure made using RNAfold provided by Vienna package. (B) Secondary structure representation of mmCNN, in main article only stem information was used since other secondary structures can be expressed using stem information only.
