# Supplementary material for: Prediction of binding property of RNA-binding proteins using multi-sized filters and multi-modal deep convolutional neural network
Source: PLoS One. 2019 Apr 26;14(4):e0216257. doi: 10.1371/journal.pone.0216257 (PMC6485761; doi:10.1371/journal.pone.0216257)
Supplement: S7 Fig — For optimizer selection 5 sets of weight initialization range and 5 running rates for Adadelta, Adam, Adagrad, and Rmsprop optimizers. (PDF) [file pone.0216257.s007.pdf]

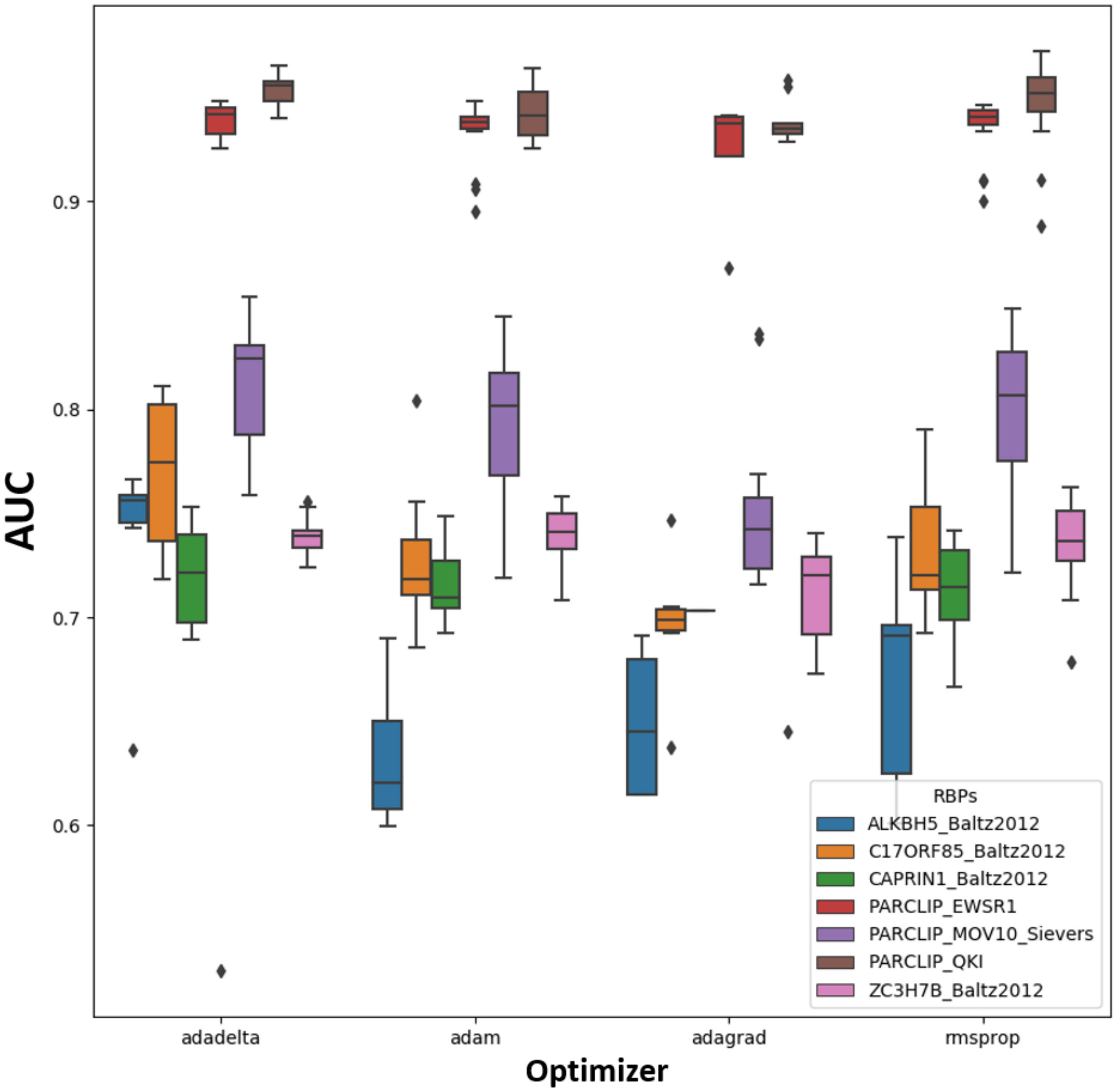

S7 Fig. mmCNN model optimization using 7 sample RBPs. For optimizer selection 5 sets of weight initialization range and 5 running rates for Adadelta, Adam, Adagrad, and Rmsprop optimizers.
