# Supplementary material for: Prediction of binding property of RNA-binding proteins using multi-sized filters and multi-modal deep convolutional neural network
Source: PLoS One. 2019 Apr 26;14(4):e0216257. doi: 10.1371/journal.pone.0216257 (PMC6485761; doi:10.1371/journal.pone.0216257)
Supplement: S1 Table — (PDF) [file pone.0216257.s008.pdf]

Type of weight	Set1	Set2	Set3	Set4	Set5
Convolution	1e-6, 1e-3	1e-5, 1e-3	1e-4, 1e-2	1e-3, 1e-2	1e-3, 1e-2
Dense	1e-5, 1e-2	1e-4, 1e-2	1e-4, 1e-2	1e-3, 1e-2	1e-2, 1e-1

S1 Table. Five different sets of convolution and dense weight initialization ranges.
