# Supplementary material for: Prediction of binding property of RNA-binding proteins using multi-sized filters and multi-modal deep convolutional neural network
Source: PLoS One. 2019 Apr 26;14(4):e0216257. doi: 10.1371/journal.pone.0216257 (PMC6485761; doi:10.1371/journal.pone.0216257)
Supplement: S2 Table — (PDF) [file pone.0216257.s009.pdf]

S1 Table. Five different learning rates (lr) tested for four optimizers.

Optimizer	lr1	lr2	lr3	lr4	lr5
Adadelta	1.0	0.5	0.1	0.05	0.01
Adam	0.001	0.0005	0.0001	0.00005	0.00001
Adagrad	0.01	0.005	0.001	0.0005	0.0001
Rmsprop	0.001	0.0005	0.0001	0.00005	0.00001

For learning rate, we started our experiment with default value proposed by Keras team, which is lr1 in (Table S2). Additionally, four different learning rate in decreasing order were tested.
